# Determiner-Number Specification and Non-Local Agreement Computation in L1 and L2 Processing

**DOI:** 10.1007/s10936-022-09864-w

**Published:** 2022-03-24

**Authors:** Yesi Cheng, Jason Rothman, Ian Cunnings

**Affiliations:** 1 School of Psychology and Clinical Language Sciences, The University of Reading (Whiteknights Campus), Reading, RG6 7BE UK; 2 UiT, The Arctic University of Norway, Universidad Nebrija, Norway, Spain

**Keywords:** Non-native sentence processing, Non-local agreement, Determiner-number specification, Self-paced reading

## Abstract

The present study employed a self-paced reading task in conjunction with concurrent acceptability judgements to examine how similar or different English natives and Chinese learners of English are when processing non-local agreement. We also tested how determiner-number specification modulates number agreement computation in both native and non-native processing by manipulating number marking with demonstrative determiners (*the* versus *that/these*). Results suggest both groups were sensitive to non-local agreement violations, indexed by longer reading times for sentences containing number violations. Furthermore, we found determiner-number specification facilitated processing of number violations in both native and non-native groups in an acceptability judgement task only, with stronger sensitivity to violations with demonstrative determiners than those with bare determiners. Contrary to some theories that predict qualitative differences between native and non-native processing, we did not find any significant differences between native and non-native speakers, despite the fact that the Chinese speakers of English had to process a novel linguistic feature absent in their native language.

## Introduction

What underlies the similarities and differences with respect to grammatical acquisition and processing between native (L1) speakers and second language (L2) learners has been strongly debated. In research in L2 acquisition, so-called representational deficit accounts, such as the Failed Functional Features hypothesis (Hawkins & Chan, 1997), claim that adult L2 learners have limited recourse to acquire native-like mental representations for L2 morphosyntactic features absent in their L1. Updated versions of such accounts, such as the Interpretability Hypothesis (e.g., Hawkins & Casillas, 2008; Tsimpli & Dimitrakopoulou, 2007), propose that it is only uninterpretable (grammatical) features that are not instantiated in the L1 that become inaccessible in adult L2 acquisition, such as number agreement on verbs. Alternatively, other L2 acquisition theories, such as the Full transfer/Full access (Schwartz & Sprouse, 1996), maintain that L2 learners can eventually acquire novel L2 features by modifying the interlanguage until it becomes L1-like. Similar debates are found in research examining real-time L2 processing. Processing accounts, such as the Shallow Structure Hypothesis (SSH; Clahsen & Felser, 2006, 2018) predict that L2ers, irrespective of L1 background, rely mainly on non-syntactic information and, as a result, construct less detailed syntactic representations of complex structures, such as non-local linguistic dependencies, compared to L1ers. Alternatively, others argue that proficient L2ers can compute syntactic structures in the same way as L1ers, though quantitative rather than qualitative differences between L1ers and L2ers may arise from issues related to memory retrieval interference, cognitive efficiency and/or participant-level individual differences (e.g., Cunnings, 2017; Hopp, 2014; Kaan, 2014; Lempert, 2016; McDonald, 2006; Sagarra & Herchensohn, 2010; Tanner et al., 2014a).

Subject-verb agreement, as in (1) and (2), forms an important test case to inform the abovementioned theoretical debates. In (1/2), the verb (“are”) must agree with the sentential subject in number. As a result, both (1) and (2) contain an agreement violation due to the number mismatch between the subject and the verb. (1) contains local agreement, as the sentence subject and verb are directly adjacent. However, (2) is a more complex structure, termed a “non-local dependency”, as the verb (“are”) is not adjacent to its subject (“The park”) because of the intervening noun phrase (intervening NP; “the flats”) in the prepositional phrase (“near the flats”).

*The window are clean (local agreement violation)
*The park near the flats are huge (non-local agreement violation)

Representation deficit accounts predict that difficulty should arise when an L2er attempts to acquire a novel grammatical feature. As a result, agreement should not be fully acquirable by an L2er whose L1 does not instantiate the relevant features. Meanwhile, the SSH emphasises that L2ers may under-utilise structural information during parsing, which may give rise to processing difficulty with complex syntactic structures such as non-local agreement. Note that representational deficit accounts predict difficulty only in L2ers where the L1 does not have agreement, while the SSH predicts non-local agreement processing may be generally difficult in all L2ers. Regardless, both theories assume that subject-verb agreement should cause difficulty to L2ers from non-agreement backgrounds (e.g., speakers of Chinese, Korean and Japanese). We focus on the processing of non-local agreement in this study due to the importance of non-local dependencies in assessing the potential ‘shallow’ nature of L2 processing. Although representational deficit accounts do not make specific predictions about differences between local and non-local agreement as in (1) and (2), by examining subject-verb agreement our study provides a test-case of these accounts as well. In contrast to these theories, other accounts would predict that all L2ers can acquire and process agreement similarly to L1ers given enough exposure to the L2 (e.g., Hopp, 2014; McDonald, 2006; Schwartz & Sprouse, 1996).

Subsequently, a substantial number of studies have investigated agreement processing, especially with L2 speakers of L1s that do not license agreement features, to adjudicate between these competing accounts (e.g., Alemán Bañón et al., 2017; Armstrong et al., 2018; Chen et al., 2007; Jiang, 2004, 2007; Lim & Christianson, 2015; Ojima et al., 2005; Tanner et al., 2012). While L2ers whose L1 has agreement have been reported to show native-like processing patterns (e.g., Frenck-Mestre et al., 2008; Tanner et al., 2013; Tanner et al., 2014a), findings from L2ers whose L1 does not have agreement have been mixed. For example, some studies suggest similar patterns between L1 and L2ers (e.g., Lim & Christianson, 2015), while others indicate problematic agreement computation in L2ers, indexed by insensitivity to agreement violations (e.g., Jiang, 2004). Therefore, whether subject-verb agreement computation is particularly problematic to L2ers without agreement in the L1 still remains unclear and requires further investigation.

Recently, some findings have shown that how number is marked on determiners modulates sensitivity to agreement violations in L1 and L2 processing, though in different directions. For example, while Tanner and Bulkes (2015) found a stronger sensitivity to violations when the number feature of the subject was double marked on both the determiner and the NP (“many cookies”), compared to when a bare determiner was used (“the cookies”), Armstrong et al. (2018) observed a reverse pattern in Chinese L2ers of English, with reduced sensitivity to violations following double number marking. However, these studies tested local agreement only, and this phenomenon has not been examined elsewhere in contexts of non-local agreement thus far.

(3) *The/Many cookies tastes the best when dipped in milk.

To extend on prior findings, we used a self-paced reading paradigm with concurrent acceptability judgements to measure: (i) sensitivity to non-local agreement violations like (2) in English L1ers and Chinese L2ers of English and (ii) how double number marking from determiner-number specification influences sensitivity to violations in L1 and L2.

### Agreement Processing in L1

A large amount of L1 literature has studied processing of number agreement violations during real-time comprehension, using various methods (e.g., Alemán-Bañón & Rothman, 2019; Dillon et al., 2013; Hammerly et al., 2019; Osterhout & Mobley, 1995; Osterhout et al., 1996; Pearlmutter et al., 1999; Shen et al., 2013; Tanner, 2011; Tanner et al., 2014a, b; Wagers et al., 2009). L1ers are consistently sensitive to agreement violations, and as a result, typically show slower reading times for sentences containing violations in comparison to sentences without violations (e.g., Dillon et al., 2013; Pearlmutter et al., 1999; Wager et al., 2009). However, in contexts where a non-local agreement violation occurs as in (2), L1ers may have difficulty with detecting such violations when the intervening NP matches the verb in number. One influential account describes this finding in terms of similarity-based retrieval interference, which predicts that the intervening NP may be retrieved as the grammatical subject as it matches the verb on number properties, despite the fact that the subject (“the park”) is the grammatical agreement controller (e.g., Dillon et al., 2013; Jäger et al., 2017; Pearlmutter et al., 1999; Shen et al., 2013; Tanner et al., 2012, 2017).

How number is specified can influence L1 agreement processing. English allows double number marking of an NP by specifying the number feature of the determiner that modifies the noun (e.g., “many cookies”). Using such number-specified determiners (e.g., quantifiers, demonstratives), number properties are more explicit compared to when using bare determiners that do not mark number (e.g., “the cookies”). In an event-related potential (ERP) study with concurrent sentence judgements, Tanner and Bulkes (2015) manipulated this factor by using stimuli like (4) to investigate whether determiner-number specification with quantifiers, as in (4c/d), would render a stronger sensitivity to local number violations compared to cases like (4a/b), without determiner-number specification.

- The cookies taste the best when dipped in milk. (Grammatical, Number Unspecified)
*The cookies tastes the best when dipped in milk. (Ungrammatical, Number Unspecified)
- Many cookies taste the best when dipped in milk. (Grammatical, Number Specified)
*Many cookies tastes the best when dipped in milk. (Ungrammatical, Number Specified)

Ungrammatical sentences like (4c/d) yielded a P600 effect compared to grammatical sentences like (4a/b). As predicted, they found a higher judgement accuracy and a larger amplitude of the P600 effect in sentences like (4d), where the plural subject NP was preceded by a number-specified determiner, compared to in (4b), where it was headed by a number-unspecified determiner. This finding indicates that determiner-number specification using the quantifier facilitates detection of local agreement violations in L1 processing. However, whether this influences non-local agreement is as yet unknown.

### Agreement Processing in L2

A large literature has examined the acquisition and processing of agreement in L2 learners (e.g., Chen et al., 2007; Jiang, 2004; Lardiere, 2007; Lim & Christianson, 2015; Tanner et al., 2012; Wen et al., 2010; White et al., 2004). Findings from these studies have failed to provide converging evidence. While some studies suggested that L2ers may struggle with subject-verb agreement in acquisition or processing if their L1 does not instantiate number agreement (e.g., Chen et al., 2007; Jiang, 2004; Lardiere, 1998a, 1998b, 2007), other research indicates that successful acquisition and native-like processing of agreement is achievable in this population (e.g., Lempert, 2016; Lim & Christianson, 2015).

Lim and Christianson (2015) investigated processing of non-local violations in Korean L2ers of English in an eye-tracking during reading experiment, using stimuli as in (5) which contained an intervening NP that is inside a relative clause rather than a prepositional phrase like (2). Besides grammaticality, they also manipulated the number properties of the intervening NP so that it either matched or mismatched the verb on number. Their results found that, similar to L1ers, L2ers showed longer reading times for (5c) and (5d) compared to (5a) and (5b), suggesting that they detected agreement errors in both ungrammatical conditions irrespective of number match. In addition, like the L1 counterparts, L2ers showed shorter reading times for (5c), where the intervening NP matched the verb in number, than (5d) when it did not. This was taken to indicate that both groups were affected by similarity-based interference. In summary, Lim Christianson (2015) argued that Korean L2ers process subject-verb agreement similarly to L1ers.

- The teachers who instructed the students were very strict. (Grammatical, Match)
- The teachers who instructed the student were very strict. (Grammatical, Mismatch)
*The teacher who instructed the students were very strict. (Ungrammatical, Match)
*The teacher who instructed the student were very strict. (Ungrammatical, Mismatch)

Jiang (2004) examined processing of non-local agreement in Chinese L2ers of English in a self-paced reading experiment. The results, however, as opposed to Lim and Christianson (2015), suggested that L2ers lacked sensitivity to morphologically marked number information in non-local agreement as they, unlike the L1ers, failed to show significantly longer reading times for sentences containing violations than grammatical sentences. However, differences in the statistical analyses conducted may in part explain these discrepancies. Specifically, Jiang (2004) did not test for a statistical interaction between the L1 and L2 groups, and instead drew conclusions based on a significant difference found between grammatical and ungrammatical conditions in the L1 group that was not significant in the L2 group. While the relevant L2 comparison did not reach statistical significance, the Chinese group did show numerical trends that were in the same direction as the L1 group. As the crucial group by grammaticality interaction was not tested, it is difficult to be certain that the L1 and L2 groups did indeed behave differently here, and any conclusions about group differences need to be considered with caution. Furthermore, the intervening NP in the stimuli used in Lim and Christianson (2015) was in a relative clause whereas in Jiang (2004) it was inside a prepositional phrase. This could have contributed to the cross-study difference as previous findings have indicated that interference from the intervening NP may become stronger when having a prepositional phrase rather than a relative clause (e.g., Tanner, 2011; Tanner et al., 2012).

Another L2 study on non-local agreement is Chen et al. (2007), who also tested Chinese L2ers of English using a similar design to Lim and Christianson (2015), but with ERPs. Unlike Jiang (2004) and Lim and Christianson (2015) who only used a reading comprehension task to check if participants paid attention to the experiment (e.g., “Did the teacher instruct the student?”), Chen et al. (2007) employed a grammaticality judgement task during online reading to tap into L2ers’ integrated linguistic knowledge (e.g., “Is this sentence grammatical or ungrammatical?”). Even though the judgement data showed the Chinese L2ers detected agreement violations during online reading, reflected by high judgment accuracy, the ERP responses suggested that they employed a distinct neural process, a late negative shift, from L1ers who exhibited a P600 effect. Therefore, the results were taken to indicate that Chinese L2ers cannot process agreement in a native-like way.

We are aware of only two studies that have examined how determiner-number specification influences agreement processing in L2 processing. Wen et al. (2010) examined agreement between a demonstrative and noun within an NP (e.g., “These beautiful house*/houses”) in Chinese L2ers and Japanese L2ers of English with various levels of L2 proficiency. Their findings showed that advanced but not intermediate L2ers were sensitive to these agreement violations, suggesting that higher proficiency leads to more native-like processing. Note that as Wen et al. investigated agreement within the NP, their results are not directly related to our study, which tests subject-verb agreement. Note also that agreement between a demonstrative and noun can be considered a local dependency within the same phrase, and as such does not inform our understanding about potential ‘shallow’ processing in non-local dependencies.

Armstrong et al. (2018) tested Chinese L2ers using the same materials and design as in Tanner and Bulkes (2015) (see 4), in an ERP with concurrent sentence judgement study. Unlike English, Chinese does not license double number marking. Instead, number properties of a noun are mainly only marked using number-specified determiners (e.g., quantifiers, demonstratives), without any morphological marker (e.g., “many cookie”). Armstrong et al.’s results showed no differences between the ungrammatical conditions (4b), where a bare determiner “the” was used, and (4d), where a number-specified determiner “many” was used, in grammaticality judgement accuracy. In the ERP data, and similar to Tanner and Bulkes (2015), the Chinese learners of English showed a P600 effect for ungrammatical (4c/d) compared to grammatical (4a/b). However, unlike Tanner and Bulkes, the Chinese learners in Armstrong et al.’s study showed a smaller P600 effect for (4d) compared to (4b), suggesting a decreased sensitivity to local violations following determiner-number specification using quantifiers.

Armstrong et al. attributed the above difference to an L1 processing strategy transfer. Specifically, they argued that since number can be marked by determiners in both Chinese and English (e.g., both languages use quantifiers and demonstratives to mark number), this overlap prompted the Chinese L2ers to focus on the determiner that carries number properties of the noun (e.g., “many”), a strategy they would use when processing their L1. As a result of this strategy, Armstrong et al. argued, less attention was given to the plural morpheme “-s” on the noun, which contributed to the attenuated P600 effect. Nevertheless, Armstrong et al. added that this could also be a general L2 processing strategy given that they only tested one group of L2ers. However, note that some of the quantifiers used in their materials (e.g., “some”) can also occur with singular nouns (e.g., “Some bread is on the table”), which could have contributed to the null effect in the grammaticality judgement task and the reduced P600 effect found in their L2ers. Therefore, this effect needs to be further examined using other types of determiners that mark number unambiguously, such as demonstratives (e.g., these, those). Also, by examining the case of demonstratives, we can ascertain whether the reported effects in previous studies are attributed to quantification itself or, a more inclusive category, determiner-number specification. Meanwhile, no published studies have systematically examined the effect of determiner-number specification in contexts of non-local agreement in L2 processing.

### The Present Study

Against the aforementioned issues, we conducted a self-paced reading experiment with concurrent acceptability judgements to examine how Chinese L2ers of English process non-local agreement containing an intervening constituent in a prepositional phrase, as in Jiang (2004), directly compared to English L1ers. Findings will inform debates surrounding the extent to which native-like L2 processing is attainable in the domain of non-local dependencies and whether native-like acquisition of a novel L2 feature is possible. We also delved a bit deeper to see the extent to which specific properties of the target grammar might facilitate or otherwise hinder native-like processing. To this end, we probed whether double marking, determiner-number specification via demonstratives, modulates sensitivity to non-local agreement violations in L1 and L2 processing. Herein, the following research questions are addressed:

(i) Will Chinese L2ers of English be able to detect non-local agreement violations that contain a prepositional phrase?
(ii) Will determiner-number specification via demonstratives increase or decrease sensitivity to non-local agreement violations in English L1ers and Chinese L2ers of English?

According to the SSH, which predicts that L2ers under-utilise structural information during processing, Chinese L2ers may behave differently from L1ers, such that they may have processing difficulty with non-local agreement and be insensitive to agreement violations. Finding that L2 learners are not sensitive to subject verb agreement violations during processing would provide strong support for such theories. This finding would also be compatible with representational deficit accounts (e.g. Hawkins & Chan, 1997). Conversely, alternative accounts would predict that Chinese L2ers may behave similarly to L1ers and can acquire and process non-local agreement in a native-like way given enough exposure to the L2 (e.g., Hopp, 2014; McDonald, 2006; Schwartz & Sprouse, 1996).

Furthermore, L1 sensitivity to non-local agreement violations should be enhanced via determiner-number specification (Tanner & Bulkes, 2015), leading to higher judgment accuracy and longer reading times for violations following determiner-number specification. If our L2ers process double number marking differently to L1ers (Armstrong et al., 2018), they should exhibit a decreased sensitivity to non-local violations following determiner-number specification and hence lower judgement accuracy and shorter reading times for those sentences. Otherwise, if our L2ers can acquire and process number marking in a native-like way, higher judgement accuracy and longer reading times should be observed in sentences containing violations following determiner-number specification.

## Method

### Participants

The experiment was conducted with 40 English L1ers (mean age = 20.7) and 40 immersed Chinese L2ers of English (mean age = 25.7; mean age of acquisition = 8.2 years, range = 4–14 years). All participants were right-handed and had normal or corrected to normal vision. The L1 participants were undergraduates at the University of Reading. They received a small payment or course credit upon completion of the study. The L2ers learned English in a school setting in China where they were born and raised. They were all studying a higher education degree in the UK at the time of testing and reported their lengths of immersion experience (mean = 31.6 months, range = 5–120 months, SD = 30.1). Their English proficiency scores, as measured by a quick Oxford Placement Test (Oxford University Press, 2004), ranged from 26 to 56 out of 60 (mean = 43, SD = 6.33).

### Materials

We employed a self-paced reading task in conjunction with a concurrent acceptability judgement task to test participants’ online processing and comprehension of agreement violations in non-local linguistic dependencies. The motivation for using acceptability judgments is that they may be a better way to prompt participants to use grammatical information implicitly during online reading, compared to grammatical judgements which instruct participants to focus on grammatical information and may involve more use of explicit knowledge (e.g., Guo et al., 2009). The reading task consisted of 32 critical items like (6) with 4 experimental conditions, and 64 fillers, pseudo-randomised in a latin-square design so that each participant read a different list and only one condition of each item, and, therefore, 8 sentences per condition. The critical items manipulated grammaticality (grammatical vs. ungrammatical). Half the sentence subjects were singular and half were plural, while the critical verb was always singular (“is”), such that half the sentences were grammatical like (6a&6c) and half were ungrammatical like (6b&6d). Also, half the experimental sentences had “is” as the critical verb and half had “has”. The intervening noun was always singular so that it matched the critical verb on number properties. Number specification on the determiner (number-specified vs. number-unspecified) was also manipulated, using demonstratives. Half the items had a number-unspecified determiner (“The”) as in (6a&6b) whereas half had a number-specified determiner, demonstrative (“This/These”), as in (6c&6d). Within the items, the numbers of demonstratives “this/these” and “that/those” were equally distributed. All the critical sentences were followed by acceptability judgements where participants were asked to indicate whether the sentence they read was acceptable or not.

- The picture of the lake is so beautiful. (Grammatical, Number-Unspecified (NU)).
* The pictures of the lake is so beautiful. (Ungrammatical, Number-Unspecified (NU)).
- This picture of the lake is so beautiful. (Grammatical, Number-Specified (NS)).
*These pictures of the lake is so beautiful. (Ungrammatical, Number-Specified (NS)).

Of the filler sentences, 32 of the 64 were followed by acceptability judgements, half of which were grammatical and half ungrammatical. Some of these fillers had a similar structure to the experimental sentences but contained a plural verb (e.g., The motorbikes in the street are really cool.) to stop participants being strategic given that all the verbs were singular in the experimental items. The remaining 32 fillers were all grammatical and did not require participants to make a judgement. We adopted this procedure to minimise any task-related effects on reading behaviour based on participants seeing multiple ungrammatical sentences. The reading task was carried out in a web-based self-paced reading paradigm where sentences were presented word by word.

### Procedure

The study was conducted online, with participants completing the experiment in their own time and setting. All participants were first asked to complete a participant form and give informed consent. Following this, they were instructed to complete a self-paced reading task where they first saw a row of dashes that covered up the sentence to-be-read and then progressed with reading at their own pace by pressing the space bar to uncover one word at a time. Participants were told to read as naturally as they could and to make sure they understood the sentences. After all experimental sentences and half of the fillers, a question “Acceptable or unacceptable?” appeared on a different screen. As such, participants would not know until after finishing each sentence whether or not it would be followed by a judgement question. Participants had to judge whether the sentence was acceptable or not by pressing 1 (acceptable) and 2 (unacceptable) on the keyboard. Before the actual experiment, participants familiarised themselves with the procedure by completing several practice trials. Finally, the Chinese speakers of English completed the proficiency test.

### Data Analysis

Reading times were calculated at two regions of text as underlined in (6). The critical region consisted of the critical verb (“is”), while the spillover region contained the word after the critical verb (“so”). Datapoints containing reading times less than 100 ms or over 10,000 ms were removed, accounting for less than 1% of the data. Such data likely index lapses in attention.

For the reading time task, the data were log-transformed to minimise skew (see Vasishth & Nicenboim, 2016) and analysed using mixed-effects models (Baayen et al., 2008) that included sum coded (−1/1) fixed effects of Group (L1/L2), Grammaticality (grammatical and ungrammatical), Number Specification (number-specified and number-unspecified) and their interactions. Post hoc analyses were conducted for any further interactions. For the judgement data, correct answers were coded as 1 and incorrect answers were coded as 0. As such, a value closer to 1 indicates higher accuracy. For analysis, we used a binomial generalised mixed-effects model (Jaeger, 2008) containing the same sum coded fixed effects as the reading time data.

The models were fit using the maximal random effects model that converged (Barr, 2013; Barr et al., 2013). Random intercepts and slopes were included. By-subject random slopes included grammaticality*number specification, and by-item random slopes included group*grammaticality*number specification. When the maximal model failed to converge, we first removed the random correlations. If it still failed to converge, we then iteratively removed the random effect that accounted for the least variance until the model converged. The experimental materials, data and analysis code for our experiments is available at the Open Science Framework (OSF) website (https://osf.io/pnux2/).

## Results

**Fig. 1:**
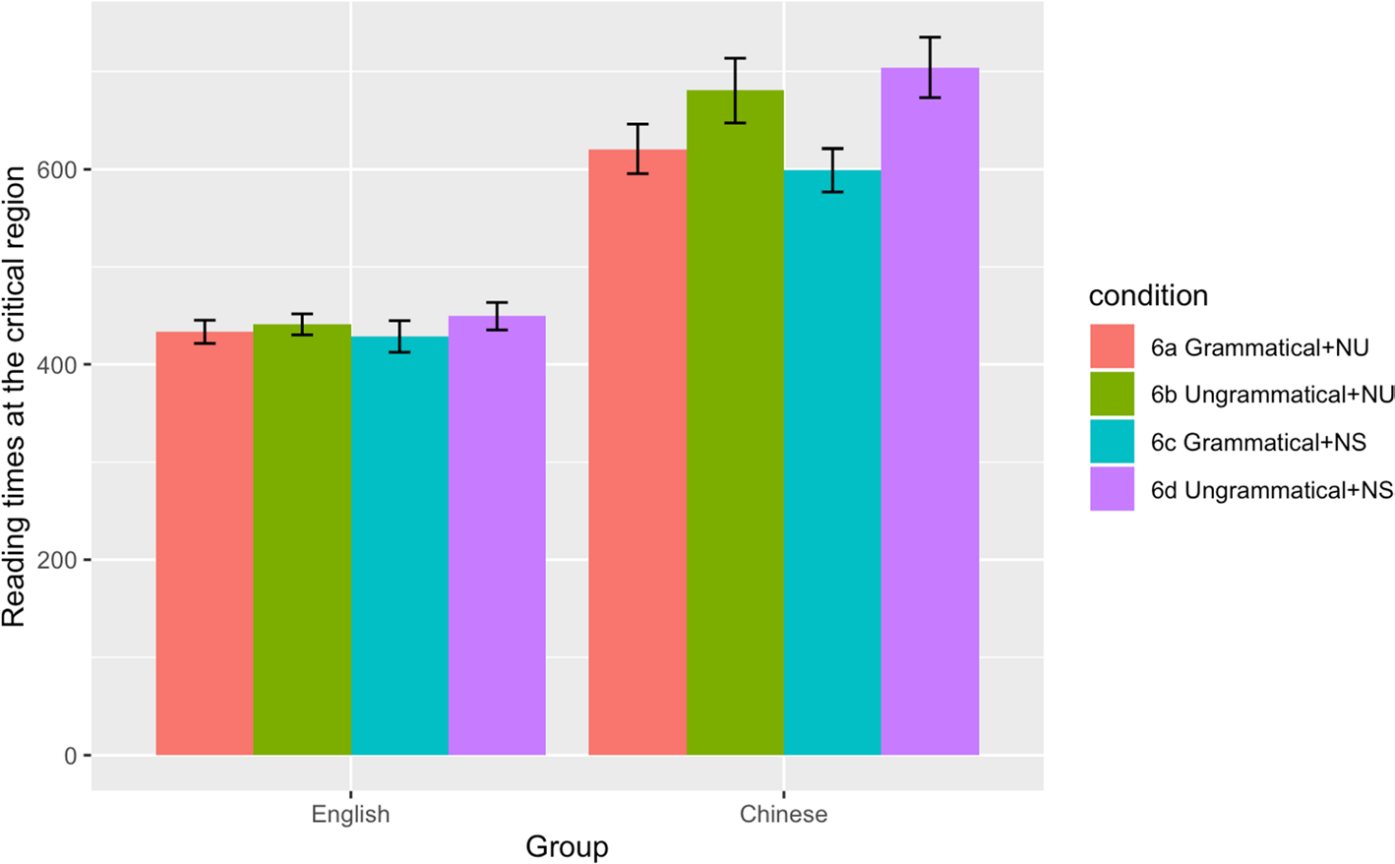
Reading times at the critical region in the L1 and L2 groups

**Fig. 2:**
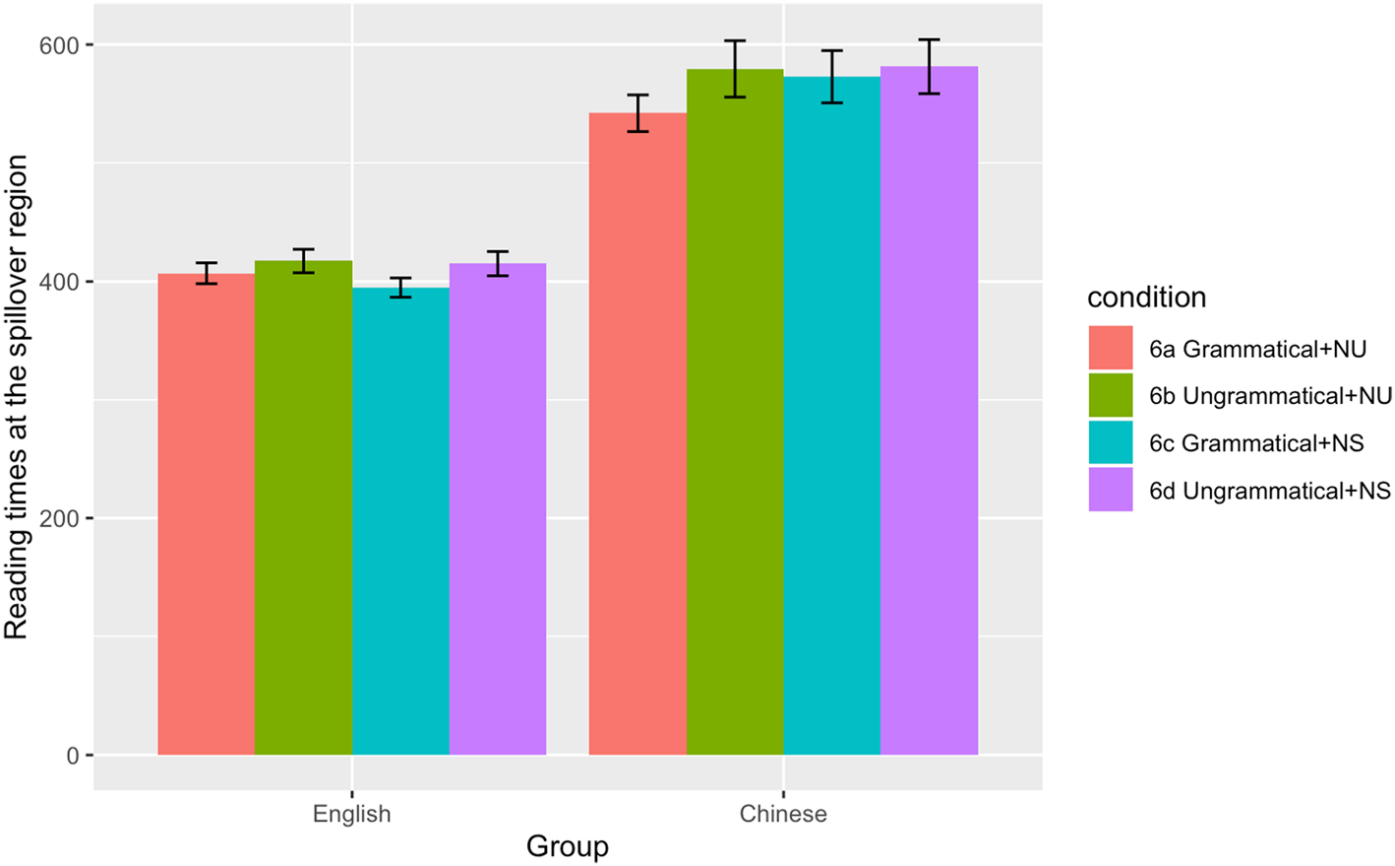
Reading times at the spillover region in the L1 and L2 groups

The summaries of descriptive and inferential statistics for both the acceptability judgement and reading time tasks are presented in Table 1 and 2. Reading time data at the critical and spillover regions are also illustrated in Figs. 1 and 2 respectively.

**Table 1:** Descriptive statistics (standard errors in parentheses) for acceptability judgements and reading times

	L1	L2
	Speakers	Speakers
*Acceptability judgements*		
Grammatical, Number Unspecified	0.92 (0.02)	0.93 (0.01)
Ungrammatical, Number Unspecified	0.88 (0.02)	0.73 (0.02)
Grammatical, Number Specified	0.84 (0.02)	0.88 (0.02)
Ungrammatical, Number Specified	0.93 (0.01)	0.81 (0.02)
*Reading times (verb)*		
Grammatical, Number Unspecified	433 (12)	621 (25)
Ungrammatical, Number Unspecified	441 (11)	680 (33)
Grammatical, Number Specified	429 (16)	599 (22)
Ungrammatical, Number Specified	449 (14)	704 (31)
*Reading times (spillover)*		
Grammatical, Number Unspecified	407 (9)	542 (16)
Ungrammatical, Number Unspecified	417 (10)	579 (24)
Grammatical, Number Specified	395 (8)	573 (22)
Ungrammatical, Number Specified	415 (10)	581 (23)

**Table 2:** Inferential statistics for acceptability judgements and reading times

	Estimate (SE)	t / z	*p*
*Acceptability judgements*			
Group	**−0.302 (0.151)**	**−2.00**	**0.046**
Grammaticality	−0.229 (0.127)	−1.81	0.071
Specification	0.031 (0.072)	0.43	0.669
Group*Grammaticality	**−0.422 (0.108)**	**−3.91**	** < 0.001**
Group*Specification	0.012 (0.071)	0.16	0.870
Grammaticality*Specification	**−0.376 (0.086)**	**−4.37**	** < 0.001**
Group*Grammaticality*Specification	0.067 (0.071)	0.95	0.345
*Reading times (verb)*			
Group	**0.162 (0.030)**	**5.37**	** < 0.001**
Grammaticality	**0.026 (0.010)**	**2.60**	**0.014**
Specification	0.001 (0.008)	0.12	0.903
Group*Grammaticality	0.012 (0.008)	1.51	0.136
Group*Specification	−0.010 (0.007)	−1.33	0.182
Grammaticality*Specification	−0.009 (0.008)	−1.17	0.247
Group*Grammaticality*Specification	−0.002 (0.008)	−0.28	0.783
*Reading times (spillover)*			
Group	**0.133 (0.025)**	**5.37**	** < .0001**
Grammaticality	0.008 (0.008)	0.96	0.338
Specification	0.000 (0.007)	0.05	0.961
Group*Grammaticality	−0.008 (0.009)	−0.93	0.357
Group*Specification	−0.010 (0.007)	−1.53	0.127
Grammaticality*Specification	−0.001 (0.007)	−0.07	0.947
Group*Grammaticality*Specification	0.004 (0.007)	0.49	0.624

Values of the effects that reached statistical significance are marked in bold

### Acceptability Judgment Results

The judgment accuracy of the fillers was 0.93 for the L1 group and 0.8 for the L2 group (all participants scored above 0.66), indicating that participants paid attention to the experiment. The overall accuracy across all four conditions was 0.89 in the L1 group (SD = 0.31) and 0.84 in the L2 group (SD = 0.37).

The statistical analysis revealed a significant Group main effect, showing that the overall accuracy was higher in the L1ers than the L2ers. This main effect further interacted with Grammaticality. Post-hoc analyses indicated that the two groups were significantly different in terms of judgement accuracy of the ungrammatical sentences only (ungrammatical estimate = -0.66, SE = 0.17, z = -3.97, *p* < 0.001; grammatical estimate = 0.11, SE = 0.21, z = 0.54, *p* = 0.588), with the L2 group having a lower accuracy in the conditions containing agreement violations compared to the L1 group. The two-way Grammaticality by Number Specification interaction was also significant, in the absence of any further significant interactions with group. Follow-up analyses revealed that Number Specification effect was significant in both grammatical sentences (estimate = 0.40, SE = 0.13, z = 3.12, *p* = 0.002) and ungrammatical sentences (estimate = -0.31, SE = 0.09, z = -3.38, *p* < 0.001), but in different directions. While accuracy for sentences containing number-specified determiners was lower than those containing number-unspecified determiners in grammatical conditions, it was higher for ungrammatical conditions.

### Reading Time Results

At the critical region, the results indicated a significant main effect of Group without any further interactions, suggesting that the L2ers had longer reading times than the L1ers. The main effect of Grammaticality was also significant, showing longer reading times spent on sentences containing agreement violations than the grammatical conditions (see Fig. 1).

At the spillover region, only the main effect of Group was significant, indicating longer reading times across conditions for the L2ers. No other significant main effects or interactions of theoretical interest were observed (all t < 1.53, all *p* > 0.127).

## General Discussion

Our results showed that although the L2ers had a lower judgement accuracy relative to the English L1ers in sentences containing non-local agreement violations, both groups were capable of detecting agreement violations in non-local linguistic dependencies, as indicated by overall accuracy in all conditions being high. Both groups also exhibited longer reading times for ungrammatical than grammatical sentences at the critical verb. Furthermore, we found a facilitation effect of determiner-number specification in the judgement data but not reading time data. We discuss the implications of our judgement and reading times data in turn below.

### Acceptability Judgements

The judgement data suggested that the L2 group exhibited good knowledge of the target linguistic feature and judged non-local agreement accurately most of the time, which is in line with previously reported L2 findings in judgement tasks (e.g., Armstrong et al., 2018; Chen et al., 2007; Ojima et al., 2005). Also, consistent with some existing L2 studies (e.g., Tanner et al., 2012), we found judgement accuracy for the ungrammatical sentences was comparatively lower in the L2ers than L1ers. This judgement error might result from interference from the singular intervening NP that has matching number features to the verb (e.g., Dillon et al., 2013; Shen et al., 2013; Tanner et al., 2012). Therefore, the lower accuracy for the ungrammatical sentences in the L2 group may be compatible with the claim that L2ers are more susceptible to interference than L1ers when processing non-local linguistic dependencies (Cunnings, 2017). The error could also result from a response bias towards grammatical/acceptable responses (e.g., Hammerly et al, 2019) as our L2ers did show a preference for more acceptable responses than the L1ers in the fillers (60% vs. 53%). Nevertheless, we do not draw any strong conclusions about the interference effect or response bias here as we neither included an ungrammatical baseline condition with a mismatching intervening NP (e.g., “The pictures of the lakes is so beautiful”), nor manipulated acceptable/unacceptable response proportions.

We also found that judgement accuracy for sentences containing non-local agreement violations increased by determiner-number specification in both groups. The higher judgement accuracy in the ungrammatical sentences with a plural demonstrative determiner reflected that the L1 and L2ers found the sentences with violations more unacceptable when the determiner is number-specified compared to when it is number-unspecified. According to Tanner and Bulkes (2015), as opposed to the number-unspecified determiner “the”, the number-specified determiner (e.g., those) allows readers to predict the number properties of the upcoming subject NP and verb as it clearly marks plurality, leading to an early and stronger prediction, and therefore a more pronounced agreement error when the prediction is violated. Alternatively, it could also be the case that the number representation of the subject becomes stronger as its number features are expressed twice with determiner-number specification, and hence violations at the verb are more pronounced. Regardless, our results suggest determiner-number specification enhances sensitivity to non-local violations in acceptability judgements, and the L1ers and L2ers were not significantly different in this regard.

However, we found a higher accuracy for grammatical sentences with a number-unspecified determiner compared to those with a number-specified determiner in both groups, indicating that our participants found grammatical sentences with a bare determiner more acceptable than those with a demonstrative. Since this difference was not present in the reading time data, we can only speculate that it is most likely spurious and might have something to do with the judgement task we employed. As we used acceptability rather than grammaticality judgements, people may have judged the sentences using non-syntactic information such as pragmatics and hence found the sentences with ‘the’ more acceptable than those with demonstratives (e.g., “this”, “those”). This could also explain why our participants found ungrammatical sentences with a demonstrative more unacceptable than those with a bare determiner.

Our results concerning the effect of determiner-number specification are aligned with previous L1 findings (Tanner & Bulkes, 2015), but inconsistent with Armstrong et al.’s (2018) L2 findings, who reported no such effect in their Chinese learners of English in a grammaticality judgement task. This could be explained by differences in the materials between studies. As mentioned previously, the use of quantifiers could have contributed to the null effect in the Chinese L2ers in Armstrong et al. (2018) as some of the quantifiers (e.g., *some*) can occur with both plural and singular nouns and corresponding verbal agreement. Conversely, our study avoided this potential confound by using demonstratives that are strictly tied to either singular (“this/that”) or plural (“these/those”) nouns and their according verbal inflections. Furthermore, the pattern in our judgement data is not consistent with the account in Armstrong et al. (2018), who reported reduced sensitivity to morphosyntactic violations following determiner-number specification in Chinese L2ers and accounted for this result using an L1 transfer strategy. Specifically, they argued that since Chinese uses lexical cues alone, such as quantifiers, to mark number, when Chinese speakers encounter double number marking using quantification and morphological cues in English, they attend more to the feature that exists in their L1 (quantification) and consequently become less sensitive to the obligatorily concurrent morphological cue (“-s”). According to their transfer-based account, we should have observed similar results (reduced sensitivity to violations) in our L2ers as Chinese also uses demonstratives to mark number. However, our results suggested a different direction of the effect. As explained earlier, the apparent reduced sensitivity in their L2ers could have arisen from their choice of quantifiers used. Given that some of the quantifiers used by Armstrong et al. could occur with singular nouns and corresponding verbal agreement, some of the violated singular verbs in their stimuli could have been processed as grammatical, which possibly reduced the overall strength of the effect in the Chinese L2ers. Therefore, we believe that Chinese L2ers do not overly rely on lexical cues from the determiner to encode number when both lexical and morphological cues are available. Instead, they utilised cues from both levels for non-local agreement computation. Our data also suggested that the effect of determiner-number specification is not limited to quantification and also applies to demonstratives.

In conclusion, our L1 and L2 participants detected number agreement violations in non-local dependencies in the judgement task. The use of demonstratives led to enhanced sensitivity to non-local agreement violations in both groups, suggesting that double number marking was similarly processed by the L1ers and L2ers.

### Reading Times

The grammaticality effect observed at the critical verb showed longer reading times elicited by the sentences containing non-local agreement violations, indicating that both L1 and L2ers noticed syntactic violations during incremental comprehension, irrespective of double number marking from determiner-number specification.

Our online findings corroborate prior L1 agreement literature (e.g., Dillon et al., 2013; Pearlmutter et al., 1999; Shen et al., 2013; Wagers et al., 2009) and some existing L2 studies, indicating longer reading times following agreement violations in L2ers with an L1 that has no agreement (e.g., Armstrong et al., 2018; Lim & Christianson, 2015). In contrast to some L2 studies that suggested qualitative differences in non-local agreement computation between English L1ers and Chinese L2ers of English (e.g., Chen et al., 2007; Jiang, 2004), our reading time data did not show any significant differences in non-local agreement processing in our Chinese L2ers as compared to English L1ers during real-time sentence comprehension. Previous research has suggested that immersive naturalistic L2 input may be associated with native-like syntactic processing (e.g., Armstrong et al., 2018; Dussias, 2003; Morgan-Short et al., 2010, 2012; Pliatsikas & Marinis, 2013), which could account for the different findings between Chen et al. (2007) and our study as their participants were tested in China whereas our participants were tested in the UK.

Recall that Jiang (2004) also tested immersed Chinese L2ers but argued that the Chinese L2ers in his study were not sensitive to non-local agreement violations during processing. However, as mentioned previously, Jiang (2004) only conducted statistical comparisons between conditions within each group and did not test for the crucial Grammaticality by Group interaction. Our study is similar to Jiang (2004) with respects to the target feature (i.e., non-local agreement with a prepositional phrase), testing paradigm (i.e., self-paced reading) and testing setting (i.e., immersion setting), though we directly compared the L1 and L2 groups. Therefore, we believe that the conflicting results between studies may result from the fact that Jiang (2004) did not conduct direct group comparisons rather than other factors, especially given that the numeral trends in Jiang’s L2 group were similar to the patterns in his L1 group. Most importantly, in contrast to what might be expected under the SSH and representational deficit accounts, in our study we did not find any significant differences between L1 and L2 speakers in terms of sensitivity to non-local agreement violations during incremental processing.

Our reading time data did not replicate the effect of determiner-number specification observed in our judgement data, which would most clearly predict a significant grammaticality by number specification interaction. There was, however, a numerical trend of this effect, which can be observed at the critical verb region in both groups, showing a larger reading time difference between grammatical and ungrammatical sentences with a number-specified determiner compared to a bare determiner (L1: 20 ms vs 8 ms respectively; L2: 105 ms vs 59 ms respectively). We are cautious in overinterpreting this effect here, but emphasise that we did not observe significant L1/L2 differences in relation to either grammaticality or number specification effects during online reading. Although our results clearly show effects of number specification in our judgement data, future research is required to further examine the effects of number specification during online processing in L1ers and L2ers. Most importantly for present purposes, despite the lack of significant effects of number specification during processing, both L1ers and L2ers demonstrated sensitivity to non-local agreement violations.

## Conclusion

We examined L1 and L2 processing of non-local agreement violations using a self-paced reading task with concurrent acceptability judgements. Both L1 and L2 groups detected non-local agreement violations in both acceptability judgements and reading times. Despite some quantitative differences in judgement accuracy for ungrammatical sentences, we did not find any signification differences between Chinese L2ers and English L1ers when processing agreement violations in non-local dependencies during online reading. Indeed, cues for number marking were processed similarly by both groups in the judgement task, with double marking from determiner-number specification facilitating detection of non-local agreement violations in L1 and L2 processing. In summary, we did not find significant differences between L1 and L2 readers in detecting number violations during processing, and as such our results do not provide support for theories that predict qualitative differences between L1 and L2 processing and acquisition.

## Data Availability

The data and materials will be made openly available on the Open Science Framework website.
